# Effect of Diabetes Mellitus Type 2 on Salivary Glucose – A Systematic Review and Meta-Analysis of Observational Studies

**DOI:** 10.1371/journal.pone.0101706

**Published:** 2014-07-15

**Authors:** Paulo Mascarenhas, Bruno Fatela, Isabel Barahona

**Affiliations:** 1 Centro de Investigação Interdisciplinar Egas Moniz, Instituto Superior de Ciências da Saúde Egas Moniz, Monte de Caparica, Portugal; 2 Serviço de Análises Clinicas - Centro Hospitalar de Setúbal (CHS), Setúbal, Portugal; University of Florida, United States of America

## Abstract

**Background:**

Early screening of type 2 *diabetes mellitus* (DM) is essential for improved prognosis and effective delay of clinical complications. However, testing for high glycemia often requires invasive and painful blood testing, limiting its large-scale applicability. We have combined new, unpublished data with published data comparing salivary glucose levels in type 2 DM patients and controls and/or looked at the correlation between salivary glucose and glycemia/HbA1c to systematically review the effectiveness of salivary glucose to estimate glycemia and HbA1c. We further discuss salivary glucose as a biomarker for large-scale screening of diabetes or developing type 2 DM.

**Methods and Findings:**

We conducted a meta-analysis of peer-reviewed published articles that reported data regarding mean salivary glucose levels and/or correlation between salivary glucose levels and glycemia or HbA1c for type 2 DM and non-diabetic individuals and combined them with our own unpublished results. Our global meta-analysis of standardized mean differences on salivary glucose levels shows an overall large positive effect of type 2 DM over salivary glucose (Hedge's *g* = 1.37). The global correlation coefficient (r) between salivary glucose and glycemia was large (r = 0.49), with subgroups ranging from medium (r = 0.30 in non-diabetics) to very large (r = 0.67 in diabetics). Meta-analysis of the global correlation between salivary glucose and HbA1c showed an overall association of medium strength (r = 0.37).

**Conclusions:**

Our systematic review reports an overall meaningful salivary glucose concentration increase in type 2 DM and a significant overall relationship between salivary glucose concentration and associated glycemia/HbA1c values, with the strength of the correlation increasing for higher glycemia/HbA1c values. These results support the potential of salivary glucose levels as a biomarker for type 2 DM, providing a less painful/invasive method for screening type 2 DM, as well as for monitoring blood glucose levels in large cohorts of DM patients.

## Introduction

Early screening of type 2 *diabetes mellitus* (DM) is essential for improved prognosis and effective delay of clinical complications associated with diabetes, and has been suggested as an important strategy to lower the incidence of this disease worldwide [1]–[3]. To date, urine and blood tests are available for screening type 2 DM. However, urine tests suffer from several drawbacks. First, increases in blood sugar levels need to be large to be detected in urine. Second, urine accumulates over time, and is therefore more difficult to collect under “fasting” conditions than blood. For these reasons, blood testing, by needle finger pricks or blood draw, remains the standard for screening, monitoring and diagnosing diabetes, despite being invasive and painful. Moreover, these inconvenient techniques perturb daily life, cause anxiety and are difficult to do in long-term diabetics due to development of finger calluses, poor peripheral circulation and risk of infection. Recent studies have focused on the development of saliva-based tests for screening or monitoring systemic diseases, including *diabetes mellitus* [4]–[6]. Saliva testing could potentially bypass the issues associated with both urine and blood tests: it is non-invasive and painless, and can be performed with ease at any time. Such an approach would be particularly useful in the young, in the elderly, and for large-scale screening or epidemiological interventions [7], [8]. However, the effectiveness of saliva-based tests is still under debate.

Several primary studies have explored the use of salivary glucose to measure glycemia, with varying success [9]. In general, salivary glucose levels in type 2 DM patients seem to be higher than in non-diabetic controls [10]–[22]; however, this finding remains controversial, as in other studies no significant differences were detected [23]–[25], or only detected in DM patients that had poor metabolic control [26], [27].

The correlation between salivary glucose and blood variables like glycemia or glycated hemoglobin (HbA1c) across type 2 DM and non-diabetic studies also shows some inconsistency in the available bibliography. Among type 2 DM patients, some studies point to a high to medium strength correlation of salivary glucose with glycemia [9], [13], [20], [21] despite other studies not finding any meaningful association [12], [24], [25], [28] or, at most, a very weak one [18] and sometimes only significant in type 2 DM patients with a bad metabolic control of the disease [11], [19]. Only a few studies have examined the correlation between salivary glucose and HbA1c, in type 2 DM patients, and found a medium strength correlation [13], [21]. Finally, in non-diabetic individuals, some studies report that salivary glucose levels do not seem to be clearly correlated with glycemia or HbA1c [18], [28], [29] although others claim the opposite [20] or, at least, show a medium strength correlation between salivary glucose and glycemia [13], [21], [30]. These results have led some authors to suggest that monitoring the salivary glucose level can be useful to evaluate the glycemic status of type 2 diabetic patients [9], [13], [20], [21], [30] and potentially to screen for early diabetes [13], [20], while others support that type 2 DM has an effect on salivary glucose but reject the idea of a consistent and direct relationship between unstimulated salivary glucose and glycemia [12], [19], [25], [28], [29].

With the latter in mind, we have performed a meta-analysis by combining published data on comparisons of mean salivary glucose levels in type 2 DM and healthy individuals, correlation studies between saliva glucose levels and glycemia/HbA1c, as well as our own unpublished results, to systematically assess whether salivary glucose can be used effectively to estimate blood glucose levels. We further discuss the potential of this approach for the diagnosis of early and late type 2 DM, and its possible use as a biomarker for diabetes or developing diabetes type 2 in large cohorts.

## Materials and Methods

The unpublished results included in this systematic review and meta-analysis came from an original cross-sectional observational study performed at our dental clinic campus. Population, saliva collection and processing, salivary glucose measurement, blood collection and measurements, and statistical analysis sections below refer to our original study.

### Population

Ethical permission for conducting this study was obtained from Egas Moniz Ethic Committee of Egas Moniz Cooperativa de Ensino Superior. Each participant signed the approved written informed consent where the purpose of the research was clearly stated; participation was entirely voluntary. Participants were divided in two different groups (Table 1): The first group consisted of 45 adult individuals of both sexes with a previous diagnosis of type 2 DM who were randomly selected from the Egas Moniz campus dental clinic patient's population, Monte de Caparica, Portugal. The second group included 16 adult individuals of both sexes without the disease (control) randomly selected from the Monte de Caparica population, Portugal. Subjects with any other pathology/disease that could affect salivary glands function or with *gingivitis* at the time of the study were not included. Other exclusion criteria were pregnancy and alcoholism. Participants were asked to fast and abstain from smoking in the night and in the morning prior to the sampling.

**Table 1:** Groups characteristics: number, average age, sex ratios, mean HbA1c, mean glycemia and poor metabolic control ratio of type 2 DM subjects.

N = 30	N	Average AGE	AGE range	Sex ratios	Mean HbA1c (%)	Mean glycemia (mg/dl)	Poor metabolic control (HbA1c>7,5%)
Type 2 DM	45	66	[27–88]	22M,23F	7.3%	167.7	40%
Control	16	60	[32–84]	7M,9F	5.6%	107.4	NA

NA: Not applicable; M: male; F: female

### Saliva collection and processing

Total unstimulated saliva was collected in the early morning from fasting subjects using the spitting method. Once the collected saliva filled at least 2 ml of a sterile standard container, it was centrifuged at 10.000 rpm for 30 minutes at 4°C. The sediment was discarded and the samples were kept on ice and measured for glucose.

### Salivary glucose measurement

Salivary glucose determination was performed with the colorimetric kit Glucose (GO) Assay (Sigma-Aldrich, Inc.) based on glucose-oxidase reaction. The standard protocol was adapted for 300 µl microplates wells and five glucose solutions with the following concentrations: 0, 5, 10, 15 and 20 µg/ml being used as standards. Absorbance values were measured at 540 nm (Bio-Rad microplate reader M680) as suggested in the manufacturer's protocol.

### Blood collection and measurements

A venous blood sample was obtained from each subject immediately after saliva collection and kept in a tube containing EDTA. Glycemia and HbA1c were measured through the following methods: Glycemia: electrochemical coulometry using a glucosimeter (Freestyle Precision - Abbots Diabetes Care Inc), yielding blood glucose in milligrams per deciliter (mg/dL), HbA1c: High Pressure Liquid Chromatography (D-10 Hemoglobin testing system – Bio-Rad Inc.), which gives the % of HbA1c fraction.

### Statistical analysis

The normal distribution of the variables was confirmed by Kolmogorov-Smirnov test before the use of a Student *t* test for independent samples. Summary statistics obtained from the data included the mean and standard deviation (SD), and differences between samples were considered significant when p≤0.05. Calculations were done using the statistical software SPSS version 19.0 (IBM, USA).

### Literature search strategy

We performed a systematic search of PubMed (title search), Google Scholar (title search) and http://b-on.pt (title and keyword search) for peer-reviewed articles about comparisons of salivary glucose values on healthy/type 2 DM subjects and for peer-reviewed articles containing data on the correlation of salivary glucose with glycemia or HbA1c, published and available online as full text before July 2013. A complementary search was performed within the references cited by selected articles.

#### Mean salivary glucose levels

The search terms used to screen for articles about the effect of diabetes mellitus type 2 on salivary glucose were “salivary and glucose”, “glucose and saliva”, “salivary and diabetic”, “salivary and diabetes”, “diabetes and salivary and glucose” and “diabetes and saliva and glucose”.

#### Correlations with salivary glucose levels

The search terms applied to retrieve articles relevant to salivary glucose correlation with glycemia or HbA1c issue were “saliva and blood and glucose”, “salivary and glucose and correlation”, “salivary and glucose and glycemia”, “glucose and saliva and A1c”, “glucose and saliva and correlation”, “sugar and saliva and correlation”, “glucose and saliva and association” and “sugar and saliva and association”.

### Literature screening

Papers were evaluated for their relevance first by assessing the title and second by abstract evaluation. The selected titles were then fully assessed for eligibility. Salivary glucose results in both concentration (*e.g*. mg/dl) and excretion rate (*e.g*. mg/dl/min) were accepted. Importance was given to saliva being collected as a whole (mixed saliva) after fasting for at least one hour (except for the salivary glucose/glycemia correlation data) and in an unstimulated way, since previous saliva experiments performed in our lab without these constraints had yielded inconsistent results. Abstracts and papers in languages other than English were excluded and author(s) were asked for an English copy if possible. Two studies [11], [15] with the same relevant data also present in posterior published reports [19], [22] were excluded in favor of respective posterior reports. Records were also excluded if the full-text articles were not available online and the author(s) failed to send a copy or did not supply required supplementary data. Two studies [16], [18] were rejected because the diabetic group was a mixture of type 2 and type 1 diabetic patients, potentially creating biases and heterogeneity in the diabetic sample that could skew the meta-analysis.

#### Mean salivary glucose levels

Studies were excluded unless they consisted strictly of *diabetes mellitus* type 2 data *versus* healthy controls and were about unstimulated whole saliva collected under fasting conditions, with a fasting period of at least 1 hour.

#### Correlations with salivary glucose levels

Studies were excluded unless they contained correlation data between glycemia or HbA1c and salivary glucose from unstimulated whole saliva collected from strict diabetic type 2 patients or in a healthy group of individuals. A fasting period of at least 1 hour was required prior to blood and saliva collection for studies to be included, if the correlation involved HbA1c. No fasting period was required for the inclusion of studies looking at correlations with glycemia.

### Critical evaluation of data

#### Mean salivary glucose levels

Using the approach outlined in the previous section, nine studies were selected, and combined with our own unpublished results. Means, standard deviations and sample sizes were collected from each study for the diabetic and control groups. In studies in which the diabetic group was split into two subsets, for instance controlled and uncontrolled diabetes [14], [19], with *periodontitis* and without it [22], or obese and non-obese diabetics [10], the two were pooled and combined (or composite) sample sizes, means and standard deviations were calculated. Composite standard deviations were obtained as reported by Headrick [31], through the square root of the composite variance. Therefore, in studies with subsets some characteristics of diabetes may be distributed in the diabetic group in a non-random fashion. Although those studies were not removed from the review, they were classified as studies with increased risk of bias and treated as a subgroup in meta-analysis.

#### Correlations with salivary glucose levels

Six studies were selected to assess the correlation between salivary glucose levels and glycemia (totaling seven studies with our unpublished data). Only two studies were selected from the screening for correlation with HbA1c, three after including our data. Correlation coefficients, associated significance and sample sizes were collected from each study in order to perform the meta-analysis.

### Power analysis

*Post hoc* power analysis for *t* test and ANOVA were undertaken using G*Power 3.1.5 software [32].

### Meta-analysis

Meta-analysis calculations and graphical plots, except for forest plots, were performed with R version 3.0.0 [33] specific packages described below. Forest plots and associated calculations were made with OpenMeta[Analyst] 6.7.13 program [34] from studies results after effect sizes and associated 95% confidence intervals (CI) calculations. Quantity I^2^ was measured to assess the degree of dispersion of effect sizes and the overall homogeneity statistical significance was calculated through the χ2 test [35]. All tests were two-tailed with alpha set at 0.05 except for homogeneity test whose significance level cutoff was considered to be 0.10 due to the low power of the χ2 test with a limited amount of studies.

#### Mean salivary glucose levels

Since not all reported means were using the same units for salivary glucose, a Hedge's standardized mean difference *g* was first calculated as effect size (ES). Hedge's *g* is a group difference ES index used to measure the magnitude of difference between two groups. It is resilient to variation in sample sizes, and allows for a standardized comparison across studies using different measures for the same variable. Calculation of this index is important since large *g* values indicate a better clinical applicability of the identified differences. We followed Cohen's [36] conservative conventions for Hedge's *g* effects: small ≥0.20, medium ≥0.50 and large ≥0.80. Confidence Intervals (CI) associated with each *g* ES index were used to assess the reliability of the effect, since a wide CI are an important way to evaluate the precision of a study's findings by providing a range of likely values around the obtained *g* ES. To further evaluate and discuss *g* ES results we followed Coe's interpretation table for ES [37]. For Hedge's *g* calculations and for graphical plot generation, salivary glucose means, standard deviations and sample sizes were inserted in R version 3.0.0 as raw data, and fed to the R packages ‘MAd’ for Hedge's *g* ES and CI calculation and to ‘Metafor’ to build the random effects aggregation model (DerSimonian-Laird method) followed by graphical plotting. Diabetic group allocation (with subsets, without subsets) was used as grouping factor in the model, since sample allocation heterogeneity can be an important source of bias in meta-analysis [38]. The following moderator candidates were evaluated through a mixed-effects meta-regression model: mean age difference between the two groups (type 2 DM *versus* control), mean age of the diabetic group and fasting hours prior to sample collection, and statistical power of the study. The possibility of publication bias was evaluated using a Begg's contour-enhanced funnel plot corrected with the trimfill function of R “Metafor” package.

#### Correlations with salivary glucose levels

To evaluate the global correlation strength between salivary glucose level and glycemia or HbA1c, Pearson correlation coefficients (r) and sample size values were collected from each study and used to calculate ES estimates as transformed Fisher-z coefficients and associated standard errors through the respective formulas. This is a necessary step because although r is the most commonly used strength of association ES index, calculating standard errors for such correlation coefficients is difficult. Moreover, the distribution of r becomes skewed as the population value of r deviates from zero, and converting r to Fisher's z corrects for this skew [39]. Finally, we ran our normalized Fisher-z data through the R package ‘meta’ metagen function to calculate an overall normalized Fisher-z, using a random effects model (DerSimonian-Laird method), and through the ‘metafor’ R package for graphical plot generation. The data from diabetic and non-diabetic studies were aggregated both as a whole and as subgroups in the analysis. Finally, in order to place the overall ES back in a correlation framework, we converted the aggregated Fisher-z data back to Pearson's r correlation coefficients running the R package ‘psych’ fisherz2r transformation function, and applied Cohen's conventions [36] for r effect evaluation: small ≥0.10, medium ≥0.30 and large ≥0.50. High r values indicate a stronger correlation.

To evaluate if the strength of the association between salivary glucose and glycemia/HbA1c increased with the higher values of glycemia/HbA1c typically associated with type 2 DM we applied again an r to z Fisher transformation to the correlation data and calculated respective standard errors from each study containing estimates for both diabetic and control groups. Afterwards we estimated Cohen's *q* ES statistic [36], a measure of the difference between Fisher-z results across the diabetic and control groups. Cohen's *q* ES standard errors were calculated using standard error propagation estimations from Fisher-z data standard errors. Aggregated Cohen's *q* ES were calculated as previously reported for overall Fisher-z values. To evaluate these overall ES we followed Cohen's conventions for *q* effects [36]: small ≥0.10, medium ≥0.30, large ≥0.50. If the associated CI did not overlap with zero, the effect was considered significant, as this suggests that Cohen's *q* scores are likely to represent a true difference. High *q* values indicate a meaningful difference between two correlations.

## Results

### Flow of study selection

#### Mean salivary glucose levels

**Figure 1:**
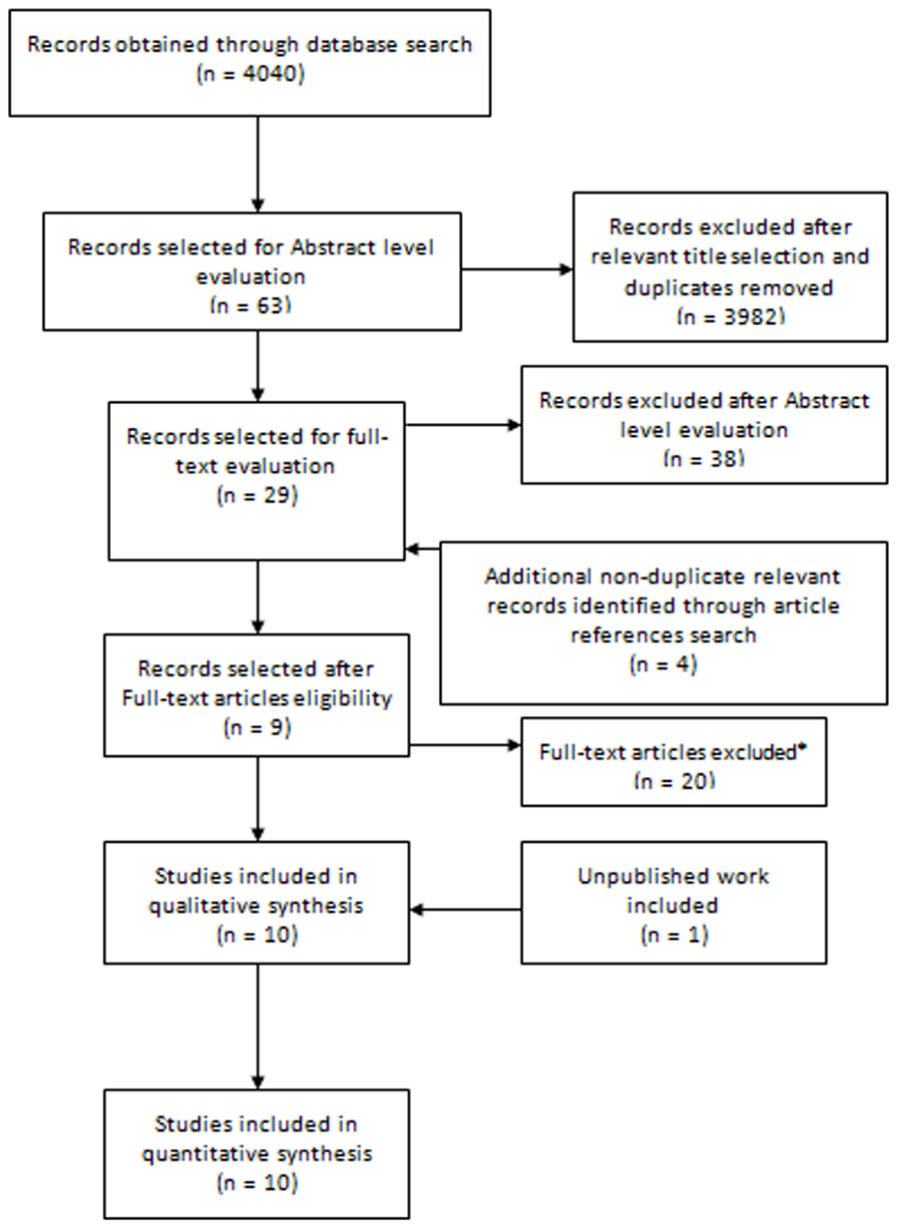
Flow of study selection for mean salivary glucose levels. *Studies were excluded unless contained salivary glucose data (means, standard deviations and sample size) obtained from strictly diabetes mellitus type 2 patients and non-diabetic controls unstimulated whole saliva collected after a minimum fast period of 2 hours. Were also excluded if the full-text article were not available and the author(s) failed in sending a copy after contact request or failed in giving back supplementary required data inexistent in the original article. Records containing data already published in other article were also excluded.

The flow of study selection for the mean salivary levels is displayed in Figure 1. Our database search initially retrieved 4040 records. After screening for relevant titles, we excluded 3982 records that were either not relevant or duplicated, and identified 63 potentially relevant studies that were assigned for abstract level evaluation. Four additional non-duplicated relevant records were identified through references found in the selected articles, and pooled with the other 63 potentially relevant studies. Among these, 9 records were selected after full-text article assessment and combined with our unpublished data, adding up to a final cluster of 10 studies.

#### Correlations with salivary glucose levels

A new electronic search was made following a similar procedure as the previous one, producing 3286 references. The examination of the titles allowed us to reject 3270 records that were not relevant, and retrieve a preliminary list of 16 potentially relevant studies. Of these, 4 were selected after abstract and full-text assessment, to which we added one non-duplicated eligible article identified in our previous search for “salivary glucose means”, and our unpublished data, resulting in a final cluster of 7 studies.

### Studies characteristics

**Figure 2:**
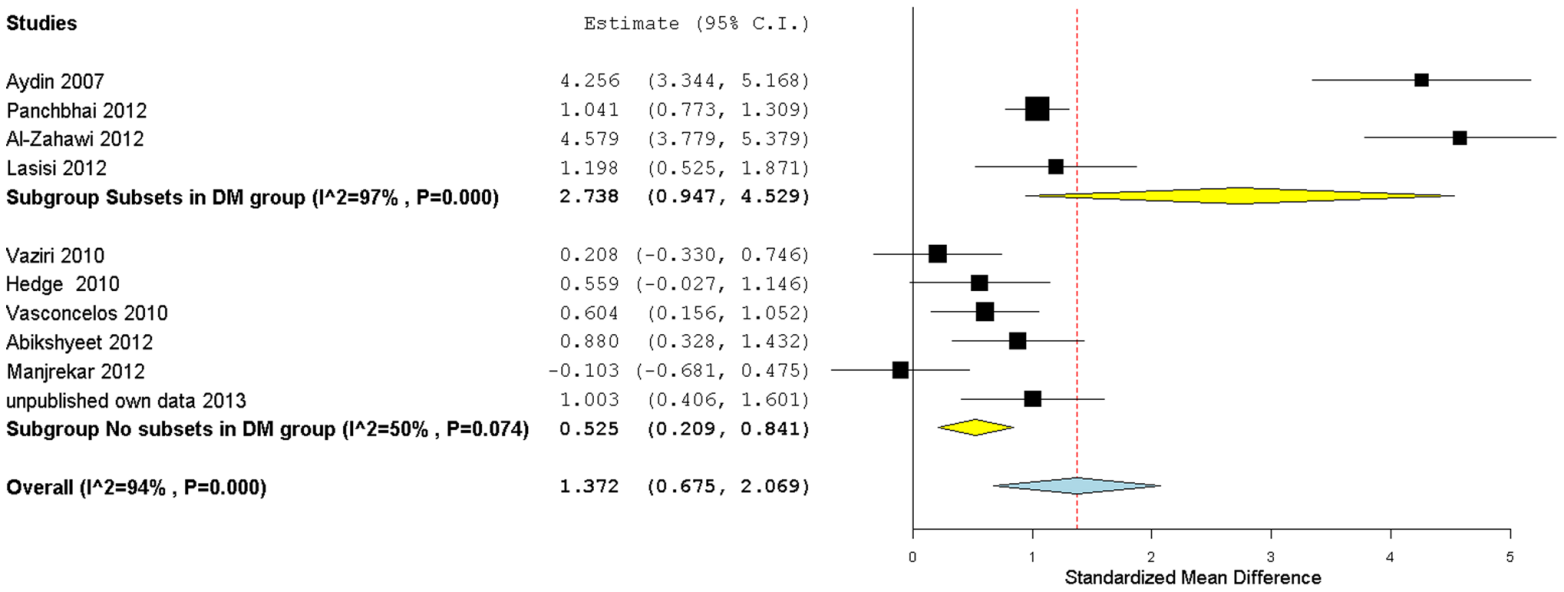
Subgroup forest plot of type 2 DM mean salivary glucose levels studies. Studies have been grouped according to the type 2 DM group allocation: with or without subsets. Hedge's *g* (standardized mean difference) effect size estimates have been calculated with 95% confidence intervals and are shown in the figure. Area of squares represents sample size, continuous horizontal lines and diamonds width represents 95% confidence interval. Yellow diamonds center indicates the subgroup pooled estimates while the blue diamond center and the vertical red dotted line both point to the overall pooled estimate. For more detailed results see Table 2 and 4.

**Figure 3:**
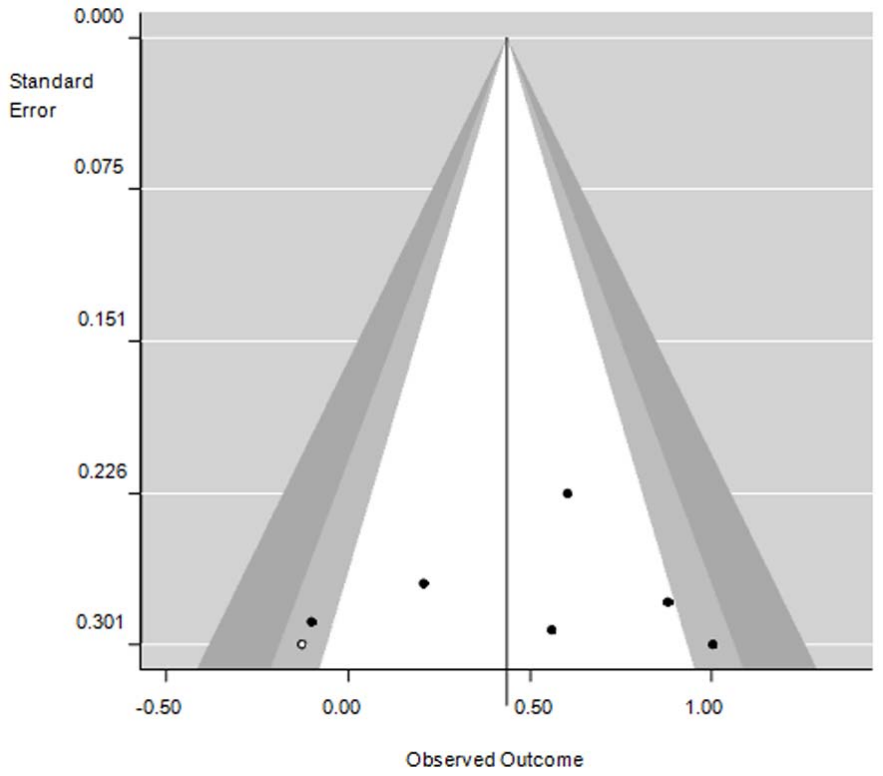
Contour-enhanced funnel plot with publication bias correction for the studies without type 2 DM subsets. Under the sensitivity analysis of the results to publication bias a trim and fill white dot was added and the plot was horizontally adjusted to maximize the dots distribution symmetry. The white region in the middle corresponds to p-values greater than 0.1, the gray-shaded region corresponds to p-values between 0.1 and 0.05, the dark gray-shaded region corresponds to p-values between 0.05 and 0.01, and the region outside of the funnel corresponds to p-values below 0.01.

**Figure 4:**
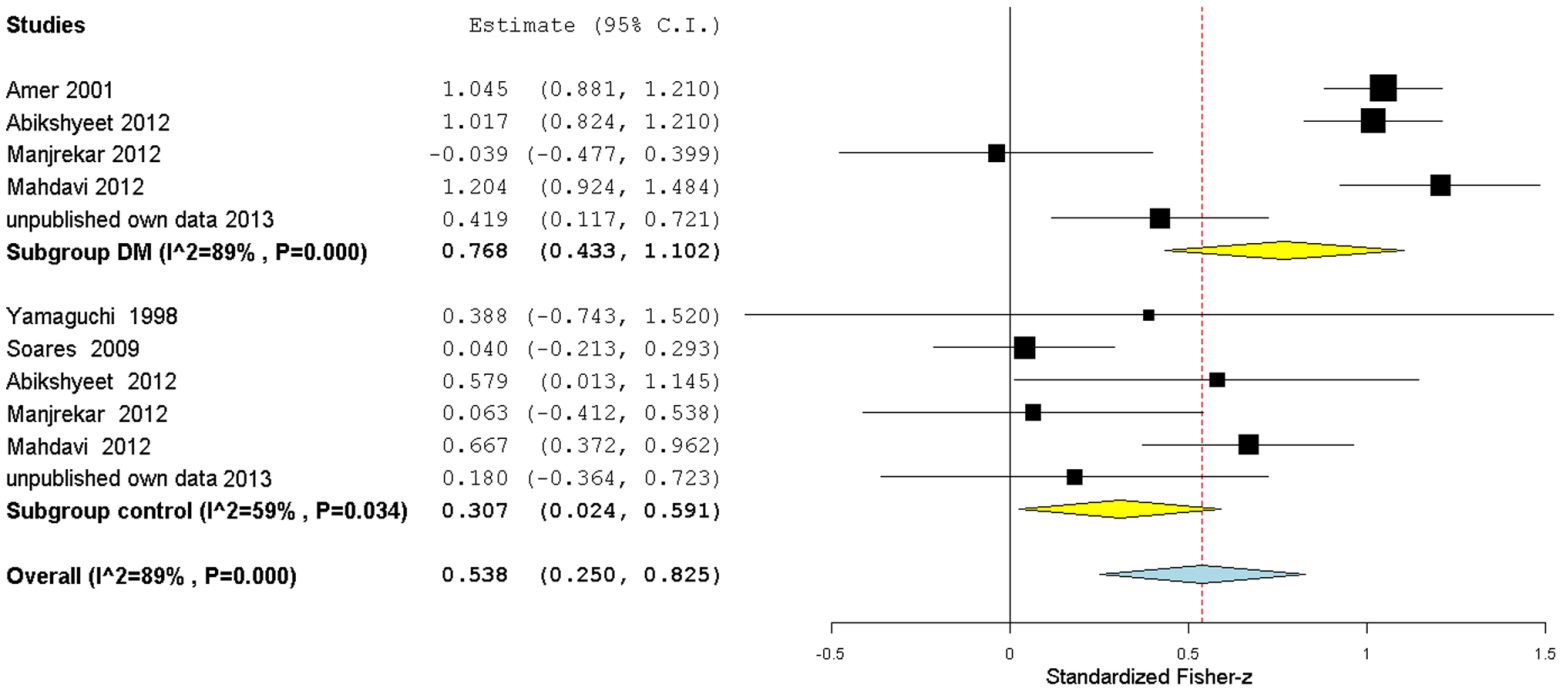
Subgroup forest plot of salivary glucose levels correlations with glycemia. Studies have been grouped according to the sample group type: type 2 diabetics or non-diabetics (control). Standardized Fisher-z effect size estimates have been calculated with 95% confidence intervals and have been aggregated (random effects model). Area of squares represents sample size, continuous horizontal lines and diamonds width represents 95% confidence interval. Yellow diamonds center indicates the subgroup pooled estimates while the blue diamond center and the vertical red dotted line both point to the overall pooled estimate. For more detailed results see Table 2 and 5.

Table 2 lists the main characteristics of the 14 studies, including our own. Ten of these were included in the meta-analysis of salivary glucose means (Fig. 2), adding up to a total sample of 580 type 2 DM patients and 297 non-diabetic controls. From these studies, only 7 were included in the meta-analysis of salivary glucose and glycemia/HbA1c correlation (Figs. 3 and 4). These studies included articles published between 1998 and 2012 and our own unpublished report. Four studies were based in India, two in Brazil and two in Iran. The remaining studies all took place in different countries: Turkey, Iraq, Nigeria, Japan, Pakistan and Portugal. The majority of the studies were conducted on patients of clinics or hospital centers, which allocated them into different diabetic groups. All the studies except one adopted exclusion criteria regarding the population sampling for type 2 DM and/or control groups, although the adopted criteria were not the same for all the studies. The majority of the studies complied with an overnight fasting period prior to sampling, two studies guaranteed only 2 hours of fasting before sampling, and another two studies reported a minimum of 90 minutes of fasting; in one study that information was not reported. Type 2 DM participant's information about diabetes duration, concomitant medications or additional disorders was missing in all studies. In two studies salivary glucose was measured through the glucose oxidase/phenol and aminophenazone method (GOD-PAP), while in the remaining studies the glucose oxidase-peroxidase method (GOD-POD) was used. Significant differences in salivary glucose between type 2 DM and healthy controls were reported in 7 studies. Among the 7 studies that examined the correlation between salivary glucose and glycemia, 5 reported results on type 2 diabetics while 6 reported results on healthy individuals. Only 3 studies reported glucose/HbA1c correlation data in type 2 diabetics, of which only 2 also examined healthy individuals.

**Table 2:** Characteristics of studies included in the salivary glucose means and correlation meta-analysis.

Data	Study	Country	Type 2 DM population	Control population	Study population exclusion criteria	Fasting on collection	Salivary glucose measurement	Outcome
Salivary glucose correlation with glycemia in healthy individuals¥	Yamaguchi 1998	Japan		The subjects in the study were six healthy young local men measured along 3 days	Affected with *pyorrhea alveolavis*.	Overnight	GOD-POD method	Found a significant low correlation between salivary glucose level and glycemia¥
Salivary glucose correlation with glycemia in type 2 DM individuals	Amer 2001	Pakistan	135 DM patients at the Diabetic OPD clinic of the Liaquat National Hospital	25 healthy local individuals	Unknown	Not reported	GOD-PAP method	A significant correlation was found between salivary glucose and glycemia in the diabetic group.
Salivary glucose in obese and non-obese** type 2 diabetics and controls	Aydin 2007	Turkey	40 patients referred by the Endocrinology Service of the Firat Medical Center equally divided in two subsets**: obese (BMI >30 kg/m2) and non-obese (BMI <25 kg/m2)	22 clinically healthy humans	Pregnancy, alcohol consumption, tobacco products (former and current), other chronic medical illness, history of drug treatment or therapy within the previous months	Overnight	GOD-POD method	Salivary glucose levels were significantly higher in obese and non-obese** diabetic subjects than in controls
Salivary glucose correlation with glycemia in healthy individuals	Soares 2009	Brazil		63 healthy patients at the dental clinic of the Federal University of Paraíba	Smokers and taking any medication, oral pathology	At least 90 min	GOD-POD method	Did not found a significant correlation between salivary glucose and glycemia in healthy individuals
Salivary glucose in type 2 diabetics and controls	Vaziri 2010	Iran	40 patients from the Hamadan Center of Diabetes Research	20 healthy subjects From Besat Hospital without medication other than vitamins or occasional analgesics	Pregnancy, alcoholism, smoking, any other chronic disease	Overnight	GOD-POD method	No significant differences in salivary glucose concentrations between type 2 diabetic patients and control subjects
Salivary glucose in type 2 diabetics and controls	Hegde 2010	India	26 DM subjects attending Kasturba medical college hospital	21 healthy subjects attending Kasturba medical college hospital	Any other systemic disease	Overnight	GOD-POD method	Salivary glucose did not differ between the two groups
Salivary glucose in type 2 diabetics and controls	Vasconcelos 2010	Brazil	40 patients from Paraiba university clinic centers	40 local healthy volunteers	Smoking, alcoholism, pregnancy, head and neck radiotherapy, autoimmune disease	At least 90 min	GOD-POD method	Salivary glucose level were significantly higher in diabetic subjects than in controls and correlation with glycemia were not significant in both DM and control groupsΔ
Salivary glucose values and correlation with glycemia data£ from two different studies** regarding type 2 diabetics and controls	Panchbhai 2012	India	Two studies with the sum of 180 patients from Wardha college and hospital equally divided in two subsets**: controlled and uncontrolled	Two studies with the sum of 90 local healthy subjects	Any other systemic disease, severe DM complications	At least 2 hours	GOD-POD method	Salivary glucose levels were significantly higher in controlled and uncontrolled diabetic** subjects than in controls, no significant differences between in uncontrolled and controlled diabetic subjects. Correlation coefficients with glycemia were low and only significant in uncontrolled DM patients £
Salivary glucose in type 2 diabetics and controls§ and correlation data with glycemia/HbA1c	Mahdavi 2012	Iran	52 patients at central laboratory of Yazd	47 subjects patients at central laboratory of Yazd	Chemotherapy or head and neck radiotherapy, dry mouth, Sjogren syndrome, heart disease, pregnancy, severe *periodontitis*, drug abuse, gland surgery.	Overnight	GOD-PAP method	Salivary glucose values were higher among diabetics than in controls. Found a significant medium strength correlation between salivary glucose and glycemia and a medium strength one between salivary glucose and HbA_1c_ in diabetics. In non-diabetics, it was found a medium strength correlation between salivary glucose and glycemia.
Salivary glucose in type 2 diabetics and controls and correlation data with glycemia/HbA1c	Abikshyeet 2012	India	106 patients newly diagnosed attending diabetic clinic	15 local healthy volunteers	Any other systemic disease, smoking, alcoholism	Overnight	GOD-POD method	Salivary glucose values were higher among diabetics than in the controls. Authors found a highly significant correlation between salivary glucose level and glycemia/HbA1c
Salivary glucose in controlled and uncontrolled type 2 diabetics** and controls	Al-Zahawi 2012	Iraq	60 patients from Erbil city health center equally divided in two subsets**: controlled and uncontrolled	30 non-diabetic patients from Erbil city health center	Any other systemic disease, severe DM complications, medication other than for diabetes	At least 2 hours	GOD-POD method	Found significant differences in salivary glucose concentrations between type 2 controlled/uncontrolled diabetic patients** and control subjects, no significant differences between uncontrolled and controlled diabetic subjects
Salivary glucose in type 2 diabetics and controls and correlation data with glycemia	Manjrekar 2012	India	23 patients from the Clinical Biochemistry laboratory of Kasturba Medical College Hospital	23 healthy controls with no family history of diabetes	history of infection in the past three months, chronic alcoholics, pregnancy	Overnight	GOD-POD method	Salivary glucose did not differ significantly between the two groups neither correlation with glycemia were meaningful
Salivary glucose in type 2 diabetics with and without *periodontitis* **and controls with* and without *periodontitis*	Lasisi 2012	Nigeria	20 diabetic patients at university of Ibadan hospital equally divided in two subsets**: with *periodontitis*, and without it	20 non-diabetics from University of Ibadan equally divided in two subsets: with periodontitis*, and without it	Not reported	Overnight	GOD-POD method	Salivary glucose level of diabetic patients was found to be significantly higher compared with non diabetic subjects irrespective of periodontal disease, no significant differences between diabetic subjects with or without *periodontitis*.
Salivary glucose in type 2 diabetics and controls and correlation data with glycemia/HbA1c	Own unpublished data 2013	Portugal	45 subjects selected from the Egas Moniz campus dental clinic, adult patient's population	16 local healthy subjects	Other pathology/disease that could affect salivary glands function or with *gingivitis* at the time of the study, pregnancy and alcoholism	Overnight	GOD-POD method	Salivary glucose values were higher among diabetics than in the controls. Found a significant medium strength correlation between salivary glucose level and glycemia/HbA1c only in diabetics.

DM: *diabetes mellitus*; GOD-POD: glucose oxidase-peroxidase method for glucose estimation; GOD-PAP: glucose oxidase/phenol +aminophenazone. *These data were not included in the meta-analysis;

**this sets of data were combined (merged) before inclusion in the meta-analysis as a subgroup of studies;

£ not included in meta-analysis, since salivary glucose/glycemia correlation global values for type 2 DM (for both controlled and uncontrolled subjects and for both studies) and controls (for both studies) were not available on the article, and the author failed to provide that information after request;

Δ not included in meta-analysis, since no correlation and significance values on salivary glucose and/or glycemia correlation were posted on the article, and the author failed to provide that information after request;

§ not included in meta-analysis, since no standard deviation data were present on salivary glucose mean results on the article, and the author failed to provide that information after request;

¥ The correlation coefficient were obtained from 3 days of measurements on six individuals.

### Data analysis

#### Mean salivary glucose levels

Initially we established which salivary glucose sampling conditions (*e.g*. minimum fasting period) were needed to obtain consistent salivary glucose measurements in our experiment, an essential step for the detection of differences in salivary glucose levels between type 2 DM and non-diabetics. Table 3 shows the salivary glucose measurements taken from 45 diabetics and 16 controls, all of them fasting individuals. These results were included in the standardized mean difference meta-analysis and show a very significant difference (p<0.01) between the salivary glucose values obtained from diabetic and control groups.

**Table 3:** Salivary glucose correlations, means and standard deviation (sd) results in type 2 DM group *versus* control.

	Type 2 DM (mean±sd) N = 45	Control (mean±sd) N = 16
Salivary glucose µg/ml	0.88±0.24*	0.56±0.47
Salivary glucose/glycemia correlation	0.40**	0.17
Salivary glucose/HbA1c correlation	0.34**	0.08

N- sample size. *significantly different from control group (p<0.01); **medium strength significant correlations (p<0.05). according to Cohen's convention [36] for r effects.

We evaluated test significance, power, Hedge's *g* and ES interpretation according to Cohen's conventions for each of the studies included in the meta-analysis (including our own, Table 4). Three studies failed to detect significant differences between the type 2 DM and control groups (p>0.1) [23]–[25], probably because they used samples of small or medium sizes, and were likely to be underpowered (*i.e*. power <0.80), which may account for the lack of statistically significant effects identified in ANOVA and t tests. The other seven studies, where significant differences were found (p<0.05), were all well-powered studies (power >0.80), with the exception of Vasconcelos study [12] with 75% of power. All studies except one [25] found an increase in salivary glucose concentration/secretion in type 2 diabetics, yielding an estimated medium to large positive effect. Furthermore, the overall positive large Hedge's *g* ES (1.37), obtained by applying a random effects model to the combined results of the studies of mean salivary glucose, also suggests a meaningful difference between the DM results and those from non-diabetic controls.

**Table 4:** Total sample size (N), significance (Sig.), power, Hedge's *g* and effect size evaluation of 10 salivary glucose observational studies in type 2 DM subjects and controls.

Study	N DM/control	Sig.	Power	Hedge's *g*	Effect size evaluation
Aydin 2007 *	40/22	<0.0001 £ ^,Δ^	99.99%	4.256	large
Vaziri 2010	40/20	0.1900 £	11.77%	0.208	small
Hegde 2010	26/21	0.2830^§^	47.54%	0.559	medium
Vasconcelos 2010	40/40	0.0360 £ ^,Δ^	74.88%	0.604	medium
Panchbhai 2012 *	180/90	<0.0001 £ ^,Δ^	99.99%	1.041	large
Abikshyeet 2012	106/15	0.0017 £ ^,Δ^	88.54%	0.880	large
Al-Zahawi 2012 *	60/30	<0.0001 £ ^,Δ^	99.99%	4.579	large
Manjrekar 2012	23/23	0.7241£	6.39%	−0.103	no effect
Lasisi 2012 *	20/20	0.0004£ ^,Δ^	96.44%	1.198	large
Own unpublished data 2013	45/16	0.0009£ ^,Δ^	96.10%	1.003	large
**Aggregated data (random effects model)**	**580/297**	<0.0001**	**99.99%**	**1,372**	**large**

Power values are relative to Hedge's *g* effect sizes for each study and aggregated data. Effect size evaluation was made following Cohen's conventions [36] for Hedge's g effects: small ≥0.20, medium ≥0.50, large ≥0.80.

£ t test significance, ^§^ANOVA significance, * combined data from type 2 DM subsets, ** omnibus random effects model significance, ^Δ^ zsignificant (p<0.05).

**Figure 5:**
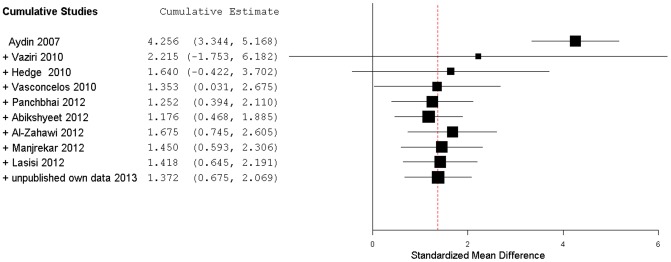
Cumulative forest plot of type 2 DM mean salivary glucose levels studies. Ten studies have been added and aggregated (random effects model). Hedge's *g* (standardized mean difference) effect size estimates have been calculated with 95% confidence intervals in a cumulative and chronological way. Area of squares represents sample size, continuous horizontal lines represents 95% confidence interval and the vertical red dotted line indicates the pooled random effect weighted estimate.

To have an historical perspective of this effect we plotted a cumulative forest plot. In Fig. 5 we can observe that earlier studies suggested a larger positive effect than the one obtained from analyzing the whole available dataset but also had lesser precision (represented by an increased CI) than the actual overall aggregated studies.

To acknowledge for the possible contribution of a selection bias to heterogeneity in the overall model regarding DM sample allocation type (with and without subsets), we performed a subgroup meta-analysis (Fig. 2) using the Hedge's *g* data from all selected studies (including our own) grouped in two clusters: one containing studies with subsets in the DM group (mixture of DM patients previously allocated to two groups) and the other one without subsets in the DM group. We verified that there was significant heterogeneity among all studies (I^2^ = 94%, p<0.10) but also within subgroups (p<0.10). The subgroup analysis (Fig. 2) showed that a substantial fraction of the heterogeneity came from studies with DM group subsets. If we look at the other subgroup of studies (without subsets), the heterogeneity value decreases to 50%. Furthermore, the latter shows an overall effect value of 0.52 (medium effect), with a [0.21,0.84] CI, which is smaller than the aggregated ES value for the combined dataset (1.37[0.67,2.07]; large effect), and also smaller than the value obtained for the subgroup with subsets of type 2 DM, which presents the largest ES (2.74[0.95,4.53]). The precision of the estimates of ES, all non-overlapping with zero CI, also differed between the subgroups, with the subgroup containing subsets of type 2 DM patients showing the widest CI. Therefore, results show that randomization in type 2 DM sampling is an important factor that affects the reliability of salivary glucose levels.

Since significant heterogeneity was detected even in the subgroup without subsets of DM patients, we further investigated the potential contribution of some candidate moderators: mean age difference between the two groups, mean age of the diabetic group, fasting hours prior to sample collection and study statistical power. To do so, we included them in a mixed-effects meta-regression model, which failed to detect a significant effect for any of them. Finally, we tested for an eventual publication bias among the subgroup of studies with no subsets of type 2 DM (Fig. 3) by applying the trim and fill correction [39]. This adjustment, with the addition of one virtual “no effect” study, decreased the overall ES in this subgroup from 0.52 (medium effect) to 0.44 (small-medium effect) and increased I^2^ from 50% to 58%.

#### Correlations with Salivary glucose levels

The results from our meta-analysis of mean glucose levels prompted us to further analyze the correlation between salivary glucose and glycemia (Table 5) or HbA1c (Table 6). Our own unpublished results, posted in Table 3, yield significant correlations (p<0.05) between salivary glucose and glycemia/HbA1c only in the type 2 DM group, and even then only a low strength correlation was found.

**Table 5:** Results on correlation (R) between salivary glucose and glycemia with Cohen's *q* effect size assessment.

Glycemia *versus* salivary glucose	Diabetic sample			Control			Cohen's *q* ±SE	Effect size evaluation
	N	R	Sig	N	R	Sig	-	-
Yamaguchi 1998	-	-	-	6	0,370	<0,05	-	-
Amer 2001	135	0.780	<0.01	-	-	-	-	-
Soares 2009	-	-	-	63	0.004	>0.05	-	-
Abikshyeet 2012	106	0.768	<0.01	15	0.522	<0.01	0.44±0.30	medium
Manjrekar 2012	23	−0.039	>0.05	20	0.063	>0.05	0.10±0.33	no effect
Mahdavi 2012	52	0.835	<0.05	47	0.583	<0.05	0.54±0.21	large
Own unpublished data 2013	45	0.396	<0.05	16	0.178	>0.05	0.24±0.32	small
**Aggregated data (random effects model)**	**361**	**0.67**	<0.0001**	**167**	**0.30**	<0.0001**	**0.35±0.14**	**medium**

Total sample size (N), correlation coefficients (R) and correlation coefficients significance (Sig). Type 2 DM effect on correlation assessment was made through Cohen's *q* statistic. ** omnibus random effects model significance.

**Table 6:** Results on correlation (R) between salivary glucose and HbA1c with Cohen's *q* effect size assessment.

HbA1c *versus* salivary glucose	Diabetic sample			Control			Cohen's *q* ±SE	Effect size evaluation
	N	R	Sig	N	R	Sig	-	-
Abikshyeet 2012	96	0.566	<0.01	-	-	-	-	-
Mahdavi 2012	52	0.516	<0.05	47	0.112	>0.05	0.46±0.21	medium
Own unpublished data 2013	45	0.341	<0.05	16	0.082	>0.05	0.27±0.32	small
**Aggregated data (random effects model)**	**193**	**0.55**	<0.0001**	**63**	**0.11**	<0.0001**	**0.40±0.17**	**medium**

Total sample size (N), correlation coefficients (R) and correlation coefficients significance (Sig). Type 2 DM effect on correlation assessment was made through Cohen's *q* statistic. ** omnibus random effects model significance.

#### Glycemia

Table 5 summarizes the correlations reported between salivary glucose levels and glycemia for the type 2 DM group: three studies found a high correlation [9], [13], [21], one study reported no correlation [25] and our own study detected a medium-strength one. In the control group, the values were on average lower, with half of the studies (including our own) reporting no correlation, while the other half found medium to weak correlations.

To assess the general ES of the correlation between glycemia and salivary glucose, we performed a subgroup meta-analysis (Fig 4) using the correlation coefficients data from all the selected studies (including our own), transformed into Fisher-z ES estimates (see Methods for more details), and grouped into two clusters: one containing studies of type 2 DM patients and the other one containing studies that included non-diabetic subjects. The overall Fisher-z results were again converted to the more intuitive Pearson's r correlation coefficients.

There was significantly high heterogeneity amount among all studies (I^2^ = 89%, p<0.10) and within the DM subgroup (I^2^ = 89%, p<0.10), while in the control subgroup heterogeneity, while still significant, was lower (I^2^ = 59%, p<0.10). The diabetic subgroup included four published reports and our own results and showed a large overall correlation (r = 0.67/z = 0.77[0.43,1.10]) between salivary glucose and blood glucose, while for the control group the combined correlation was smaller (r = 0.30/z = 0.31[0.02,0.59], medium effect). The overall correlation coefficient when both subgroups were included, was 0.49 (z = 0.54[0.25,0.82]) (medium-large effect). All these three correlation coefficients came from Fisher-z values, and had CI similar in precision and non-overlapping with zero. The larger correlation observed for the DM subgroup suggests that, in type 2 diabetic patients, increases in glycemia are paired with a stronger positive effect on the level of salivary glucose compared with non-diabetic individuals. The correlation appears to be stronger for higher values of glycemia. To further support this possibility we tested the effect of type 2 DM (*i.e*. glycemia increase) on r strength applying the Cohen's *q* statistic.

**Figure 6:**
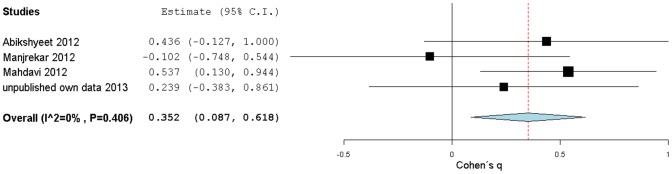
Forest plot from DM condition effect on salivary glucose levels correlations with glycemia. Cohen's *q* (standardized Fisher-z difference between diabetic and control groups) effect size estimates have been calculated with 95% confidence intervals and have been aggregated (random effects model). Area of squares represents sample size, continuous horizontal lines and diamonds width represents 95% confidence interval and the diamonds centre and vertical red dotted line indicates the pooled random effect weighted estimate. For more detailed results see Table 5.

In four studies, it was possible to calculate Cohen's *q* statistic. Cohen's *q* results were not consistent, and varied from a large ES to an absence of meaningful effect. These *q* values were used as ES estimates to build a random effects model, as shown in Fig. 6. Overall, the Cohen's *q* results estimated a significant medium strength 0.35[0.09,0.62] ES, with a non-overlapping zero CI, confirming a stronger correlation between salivary glucose and glycemia in type 2 DM relative to the non-diabetic control.

#### HbA1c

A summary of the meta-analysis of the correlation between salivary glucose and HbA1c is presented in Fig. 4. It included two published reports, in addition to our own results, for the diabetic subgroup, and only one published report, as well as our own data, for the non-diabetic control subgroup. All studies reported a medium correlation in the diabetic subgroup, while for the non-diabetic subgroup both studies failed to detect a significant correlation (Table 6).

It should be noted that, given their more straightforward physiological link, the correlation between salivary glucose and glycemia is expected to be stronger than between salivary glucose and HbA1c, and so it is unsurprising that the correlation values present in Table 5 are in average higher than the one's present in Table 6.

**Figure 7:**
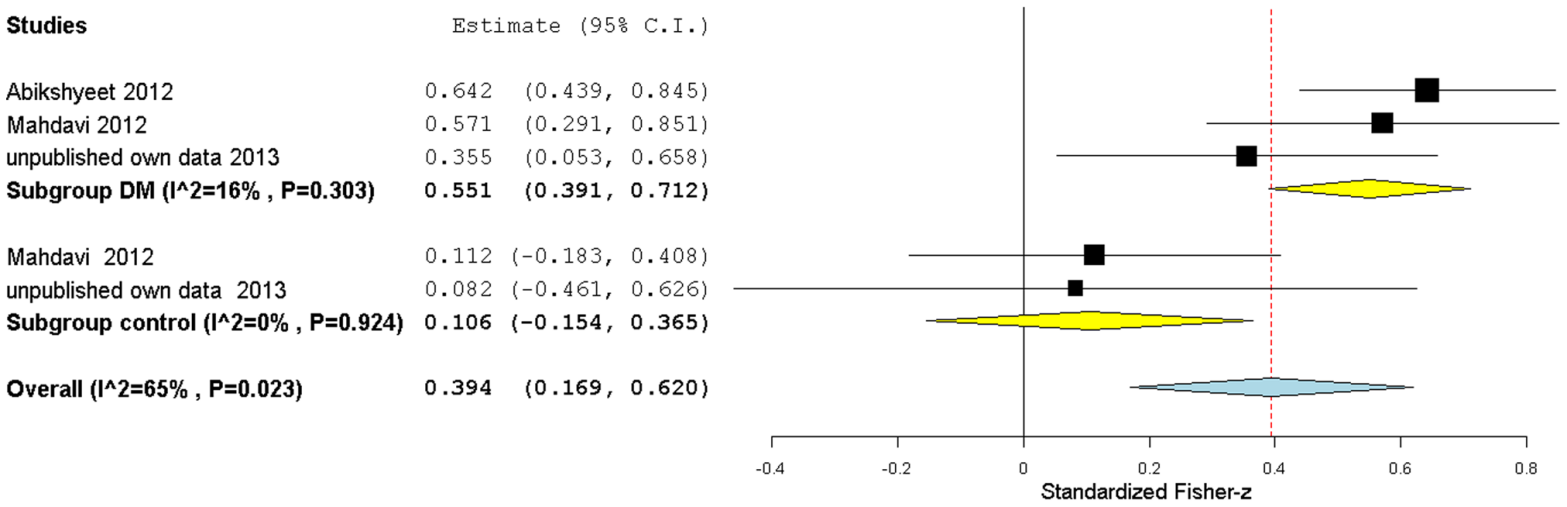
Subgroup forest plot of salivary glucose levels correlations with HbA1c. Studies have been grouped according to the sample group type: type 2 diabetics or non-diabetics (control). Standardized Fisher-z effect size estimates have been calculated with 95% confidence intervals and have been aggregated (random effects model). Area of squares represents sample size, continuous horizontal lines and diamonds width represents 95% confidence interval. Yellow diamonds center indicates the subgroup pooled estimates while the blue diamond center and the vertical red dotted line both point to the overall pooled estimate. For more detailed results see Table 2 and 6.

Once again, to estimate the overall correlation between HbA1c and salivary glucose, a subgroup meta-analysis was performed (Fig. 7) using Fisher-z transformed data from all selected studies (including our own) grouped into two clusters: one containing studies in type 2 DM patients and the other one containing studies that included non-diabetic control subjects. The meta-analysis yielded an overall medium correlation (r = 0.37/z = 0.39[0.17,0.62]) between salivary glucose and HbA1c with Fisher-z CI non-overlapping with zero. In the diabetic studies subgroup the correlation was stronger (r = 0.50/z = 0.55[0.39,0.71]) with Fisher's z CI non-overlapping with zero while a non-significant correlation (r = 0.11/z = 0.11[−0.15,0.36]) was found in the non-diabetic subgroup. Similar to what we observed for the glycemia/salivary glucose correlation, our estimates of the correlation between HbA1c and salivary glucose for type 2 DM and control subgroups (Figs. 7) suggest that, in type 2 diabetic patients, increases in HbA1c values are more strongly correlated with increases in salivary glucose than in non-diabetic control groups, where HbA1c values are on average smaller. There was overall high heterogeneity among all the studies (I^2^≈65%, p<0.10). A subgroup analysis showed that a significant fraction of the heterogeneity originated from the diabetic studies, while in the other subgroup of studies (without diabetics), the level of heterogeneity is low.

**Figure 8:**
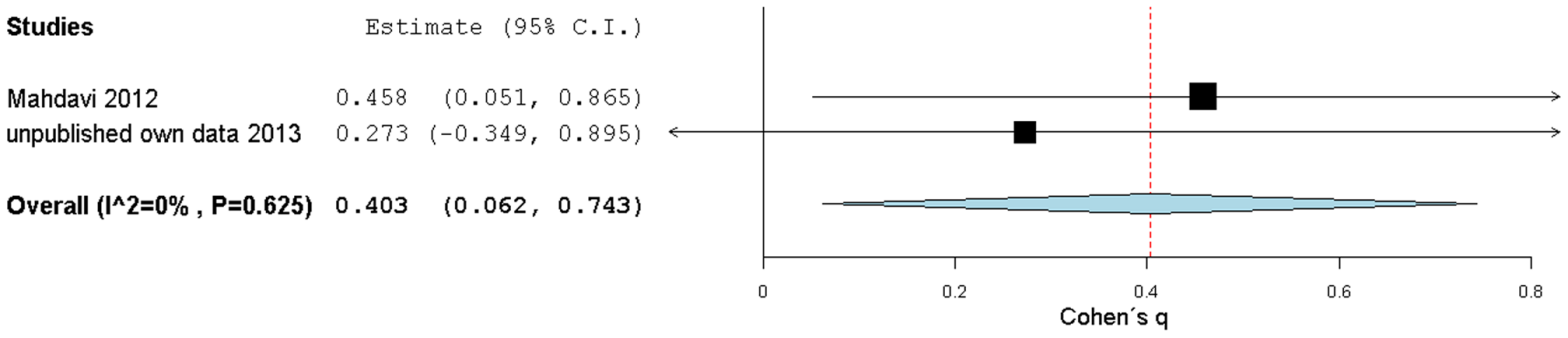
Forest plot from DM condition effect on salivary glucose levels correlations with HbA1c. Cohen's *q* (standardized Fisher-z difference between diabetic and control groups) effect size estimates have been calculated with 95% confidence intervals and have been aggregated (random effects model). Area of squares represents sample size, continuous horizontal lines and diamonds width represents 95% confidence interval and the diamonds centre and vertical red dotted line indicates the pooled random effect weighted estimate. For more detailed results see Table 6.

The correlations between HbA1c and salivary glucose for type 2 DM and control subgroups were compared using Cohen's *q* statistic in two studies, in the one from Mahdavi [21] and in our own, showed a medium and a small ES, respectively. These results were used as ES estimates to build a random effects model, which yielded an overall medium strength *q* ES of 0.40[0.06,0.74] (CI non-overlapping with zero but with low precision, Fig. 8). Cohen's *q* ES meta-analysis result shows that the correlation between salivary glucose and HbA1c mirrors the previous correlation behavior between salivary glucose and glycemia, in that correlations are stronger within type 2 DM groups (or for increased glycemia) then in the non-diabetic control groups.

## Discussion

We have performed the first systematic review of the effect of type 2 DM on salivary glucose levels, to evaluate if the increase in salivary glucose levels associated with type 2 DM is both strong and consistent enough to substantiate salivary glucose as a potential type 2 DM biomarker. This evaluation was motivated by the historical controversy surrounding this issue, with some authors defending the idea that salivary glucose based tests have the potential to become an effective and non-invasive method for diagnosing or monitoring DM, and others vividly dismissing it.

A few previous reports of salivary glucose measurements in diabetic patients included reviews of this subject, but mainly from a qualitative point of view. Furthermore, strict criteria for study inclusion were not always adopted or explicit, and differences regarding sample allocation and experimental design were generally not taken into account, potentially leading to increased selection bias. These earlier reviews (see Introduction) often disagree on the size of the difference between salivary glucose levels in type 2 DM and non-diabetics based on null-hypothesis significance testing data, and on the strength of the correlation between glycemia/HbA1c and salivary glucose, all of which are essential to assess the potential of salivary glucose for screening potential diabetic patients.

We also found that, over the years, the global Hedge's *g* ES of type 2 DM on salivary glucose changed from an initial imprecise but very high value to the current more precise and significant large value of 1.37. This progression may be a consequence of the legacy of a publication bias, or of non-random allocation of type 2 DM patients, with a recent adoption of a stricter glycemic control by type 2 DM patients, in combination with the increased precision in overall estimates achieved by increasing the number of studies included in meta-analysis. In this review, we tried to minimize study selection bias by including only published reports where saliva was being collected under similar conditions as the ones we used in our experiments.

The large overall ES value obtained from the standardized mean difference meta-analysis (1.37), according to Coe's interpretation table for standardized mean difference ES [37], means that the probability that the salivary glucose level of a type 2 DM patient exceeds the salivary glucose level of a non-diabetic is 83%, if both are chosen at random. Accordingly, the average salivary glucose score of a type 2 DM patient exceeds 91% of control salivary glucose levels values. Global ES estimates obtained from aggregated type 2 DM and non-diabetic controls studies of the correlation between salivary glucose and glycemia/HbA1c yielded consistent medium to large effects (0.49/0.37), which increased further in strength when the correlation synthesis was limited to data from type 2 DM patients (0.67/0.50), which had, on average, higher glycemia/HbA1c values. This stronger correlation within the type 2 DM subgroup suggests that the salivary glucose secretion rate increase is somehow more strongly paired with blood glucose/HbA1c on a chronic hyperglycemia background, as previously reported by Abikshyeet [13]. It is known that DM chronic hyperglycemia leads to microvasculature structural changes, as well as basement membrane alterations in salivary glands and soft oral tissues [40]–[42]. These changes result in leaky salivary glands and soft oral tissues, leading to an increase on the glucose diffusion rate from the blood to the oral cavity [13], [43]. These oral physiological changes associated with DM could therefore explain the increase in the strength of the correlation between the salivary glucose and glycemia/HbA1c in the type 2 DM subgroup.

In the non-diabetic control groups, the results of our meta-analysis of the correlation of salivary glucose levels showed a medium effect with glycemia and a non-significant effect with HbA1c. This lower effect may be related with a less permeable salivary glands/oral mucosa present in healthy individuals and/or with a possible detection limit of the employed glucose measurement technique (GOD-POD/GOD-PAP) for very low concentrations of salivary glucose. If the latter is true, saliva-based tests may be of limited use to monitor blood glucose levels in non-diabetics or diabetics with very good metabolic control of the disease.

There are other potential limitations to this systematic review. One potential source of bias includes the possible misclassification of subjects into the control or DM groups. Another issue is that our findings are based on the results of observational studies and that we can therefore not exclude the presence of confounding factors, especially relevant when ES are not very high. Studies included in our systematic review did not consistently report some important sample-related potential moderators, such as mean duration of DM, and status of glycemic control and treatment, preventing these variables from being tested as sources of heterogeneity. These factors, combined with differences in the procedures used in each study, may confound the outcome and partly explain the significant amount of heterogeneity detected in the meta-analyses of salivary glucose means and correlations. Heterogeneity may also be due to the presence of subsets (obesity, bad metabolic control or *periodontitis*) in type 2 DM groups that may change the proportion of patients with poor metabolic control. Furthermore, the lack of statistical significance found for the effect of tested moderators on the heterogeneity should be seen with caution, because of the limited statistical power provided by the small number of studies included in the meta-regression model. Up to now, no studies have examined the sensitivity/specificity of salivary glucose testing for evaluating blood glucose or even screening or monitoring type 2 DM. In the future, it will be important to define the predictive power of salivary glucose to estimate glycemia/HbA1c levels as well as its sensitivity and specificity. Despite these limitations and the reduced number of observational studies reviewed, some of them with small sample sizes, the consistency of the overall ES of salivary glucose mean and correlation with glycemia and HbA1c support the claim that type 2 DM have a positive medium to large overall effect on salivary glucose levels. This substantiates the use of this variable for type 2 DM screening, especially in large-scale studies, which could be greatly facilitated by the ease of the methodology. Furthermore, this correlation between blood and salivary glucose levels grows with increased glycemia or HbA1c, which may, in itself, be a sign of diabetes with bad metabolic control. Salivary glucose evaluation may therefore also facilitate the monitoring of metabolic status in DM patients through daily glucose self-measurements.

In conclusion, our results show that type 2 DM leads to a consistent increase in salivary glucose that remains detectable in spite of food contamination, variations in salivary flow rate or presence of local autonomic neuropathy. Our review also reports a significant overall relationship between salivary glucose concentration and associated glycemia/HbA1c values, with the correlation strength increasing as we move to higher glycemia/HbA1c values. These results, in combination with recent and old reports [13], [30], support the possible use of salivary glucose as type 2 DM biomarker. If associated with the development of sensitive portable technology to measure salivary glucose [30], [44], [45] this will allow a less painful and invasive method for type 2 DM screening or diabetic glucose monitoring, especially for studies of large cohorts. Even if at present, due to several limitations, salivary glucose *per se* may not show enough consistency to be used as an independent and autonomous DM type 2 biomarker, our results suggest that it provides valuable information, and may in the future be combined with other salivary biomarkers to create an effective high sensitivity/specificity DM type 2 large-scale screening system.

## Supporting Information

Table S1 PRISMA 2009 checklist.
(DOCX)
